# Supplementary material for: The adaptive landscape of a metallo-enzyme is shaped by environment-dependent epistasis
Source: Nat Commun. 2021 Jun 23;12:3867. doi: 10.1038/s41467-021-23943-x (PMC8222346; doi:10.1038/s41467-021-23943-x)
Supplement: Supplementary file 1 — Supplementary Information [file 41467_2021_23943_MOESM1_ESM.pdf]

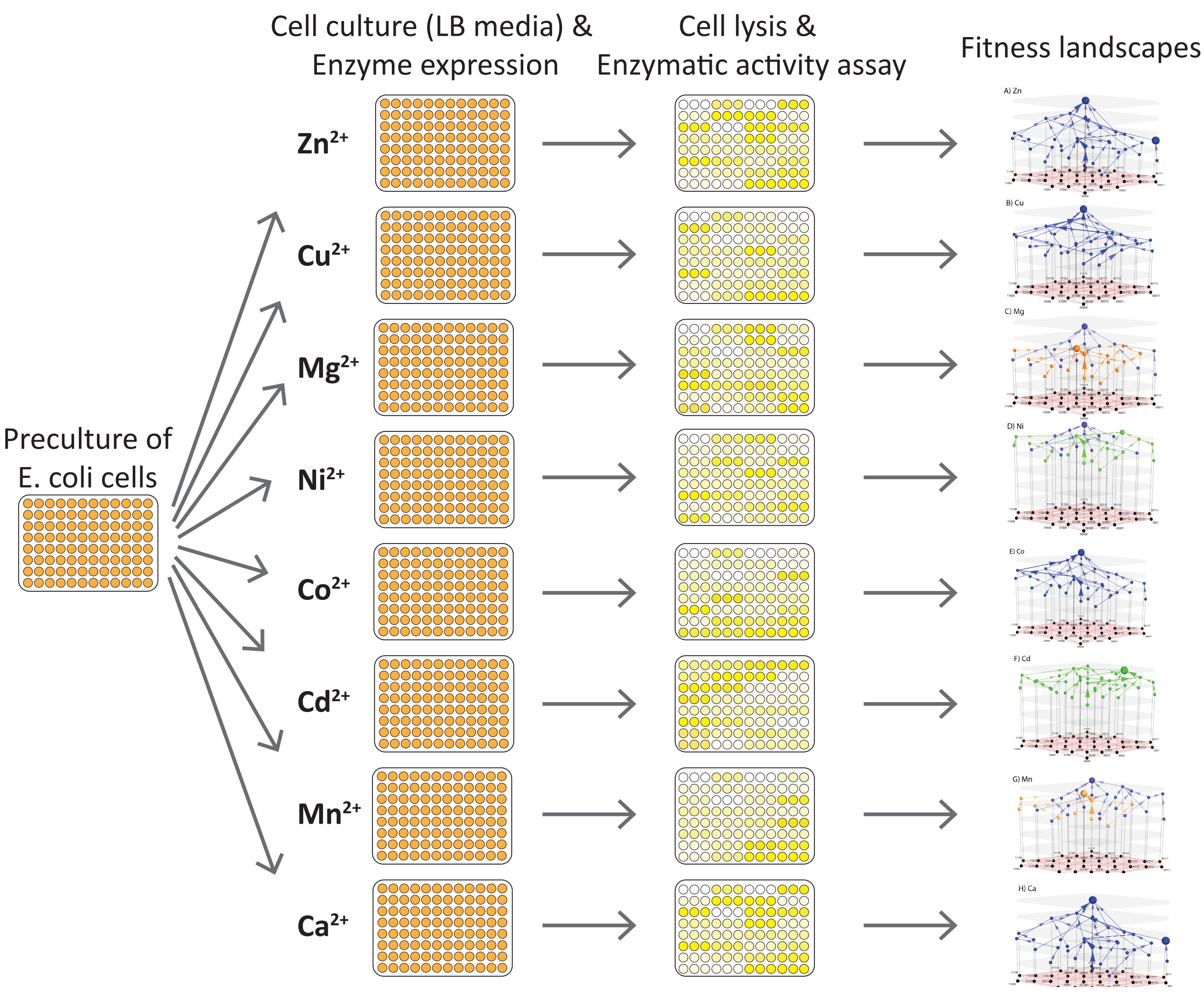

Cell culture (LB media) &
Enzyme expression
Cell lysis &
Enzymatic activity assay
Fitness landscapes
Preculture of
E. coli cells
Zn2+
Cu2+
Mg2+
Ni2+
Co2+
Cd2+
Mn2+
Ca2+
A) Zn
B) Cu
C) Mg
D) Ni
E) Co
F) Cd
G) Mn
H) Ca

Supplementary Figure 1 Outline of the experimental and analytical scheme. Enzymes were expressed in *E. coli* in the presence of 100 μM of metal supplied in the media, and lysed with 200 μM of the metal in the buffer. Lysate activities are measured using 20 μL of cell lysate and 80 μL of assay buffer containing 500 μM methyl-parathion (final concentration 400 μM) and 200 μM of metal.

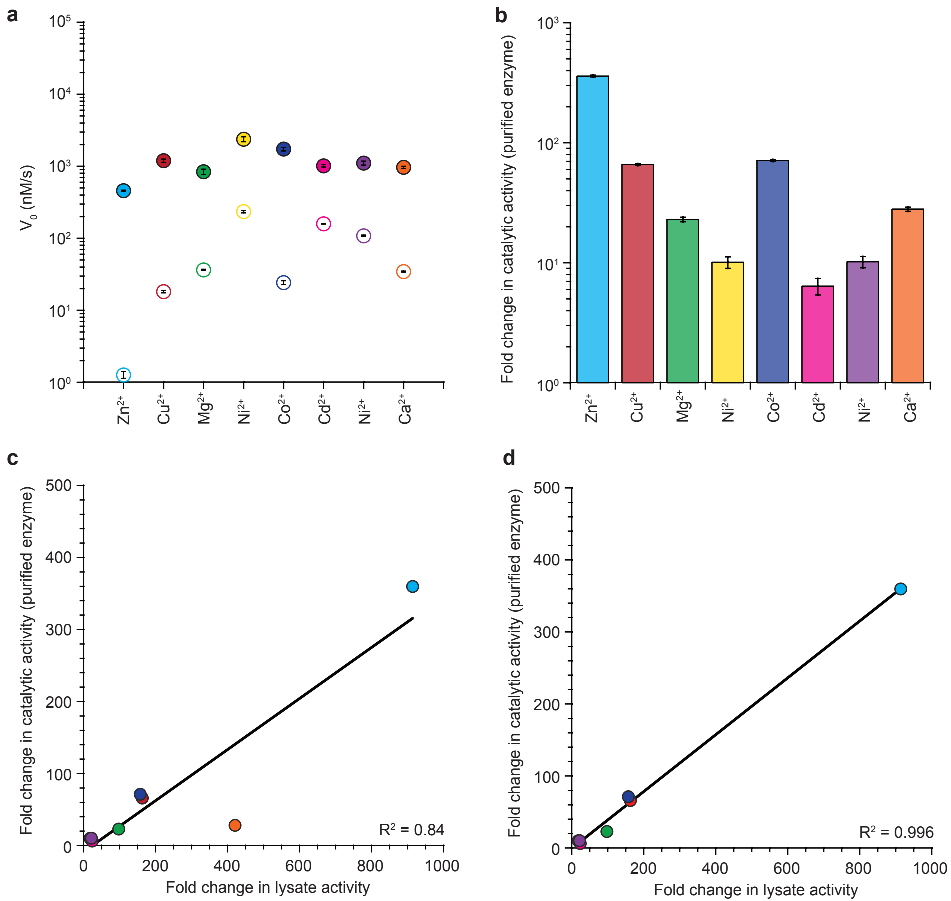

a
b
c
d
V_0 (nM/s)
10^0
10^1
10^2
10^3
10^4
10^5
Zn^{2+}
Cu^{2+}
Mg^{2+}
Ni^{2+}
Co^{2+}
Cd^{2+}
Ni^{2+}
Ca^{2+}
Fold change in catalytic activity (purified enzyme)
Fold change in lysate activity
0
100
200
300
400
500
600
800
1000
R^2 = 0.84
R^2 = 0.996

Supplementary Figure 2 a, Catalytic activities of purified enzymes. Enzymes were grown in 30 mL of 2x YT media supplied with 100 μM of metal, and purified in buffer supplied with 200 μM of the metal. The activities were assayed using 10 μL of enzyme and 90 μL of assay buffer containing 500 μM of methyl-parathion (final concentration 450 μM) and 200 μM of the metal. The activities were normalized to an enzyme concentration of 1 μM. **b,** The collective effect of all five historical substitutions (fold-change in catalytic activity between ancestral genotype and derived genotype) in each metal environment. **c** and **d**, Relation (linear correlation) between fold-change in lysate activity and fold-change in catalytic activity of purified enzymes when **c**, all metals are included and **d,** all metals except for Ca^{2+} is included.

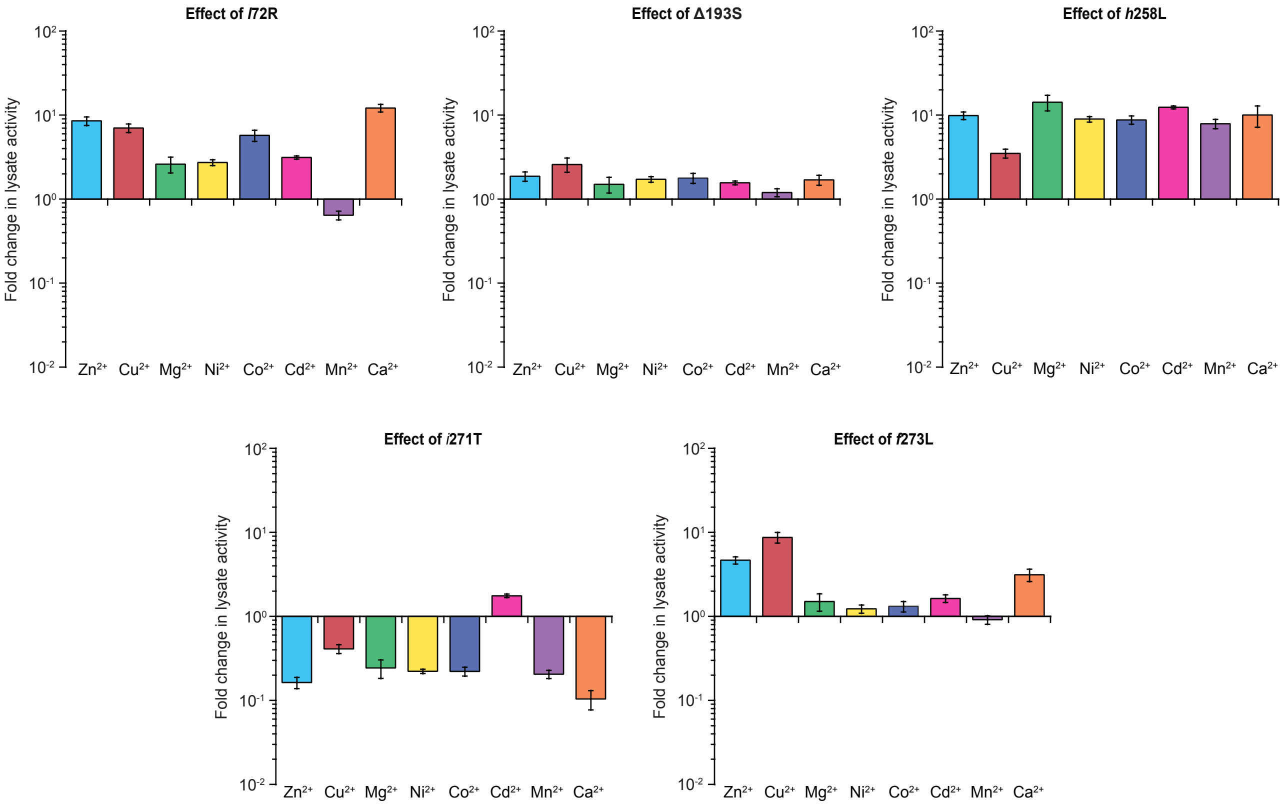
Effect of l72R
Effect of Δ193S
Effect of h258L
Effect of i271T
Effect of f273L
Fold change in lysate activity
10^{2}
10^{1}
10^{0}
10^{-1}
10^{-2}
Zn^{2+} Cu^{2+} Mg^{2+} Ni^{2+} Co^{2+} Cd^{2+} Mn^{2+} Ca^{2+}

Supplementary Figure 3 The effect of each individual substitution when introduced into the ancestral genetic background.

Shows the effect of introducing each mutation into the original ancestral background. Bars show average effect and bars show SEM, as calculated from replicate measurements.

Supplemental Tables

Terms	Zn	Cu	Mg	Ni	Co	Cd	Mn	Ca
pos72	0.295	0.188	0.083	0.070	0.297	0.035	-0.063	0.264
pos193	0.301	0.311	0.211	0.190	0.242	0.120	0.188	0.269
pos258	0.321	0.051	0.397	0.333	0.318	0.333	0.429	0.394
pos271	-0.236	-0.111	-0.151	-0.172	-0.248	-0.036	-0.103	-0.140
pos273	0.338	0.291	0.175	0.097	0.185	0.031	0.133	0.300
pos72pos193	0.179	0.161	0.132	0.064	0.113	0.018	0.094	0.090
pos72pos258	-0.129	-0.068	-0.135	-0.098	-0.069	-0.065	-0.121	-0.218
pos72pos271	0.045	0.074	0.104	0.112	0.066	0.056	0.086	0.011
pos72pos273	0.151	0.014	0.069	0.011	0.108	-0.037	0.050	0.046
pos193pos258	-0.012	-0.012	0.003	0.008	-0.001	0.025	-0.003	0.008
pos193pos271	0.021	0.037	0.015	0.027	0.014	0.017	0.014	0.027
pos193pos273	0.154	0.086	0.098	0.038	0.110	0.044	0.074	0.098
pos258pos271	0.144	0.092	0.111	0.020	0.049	-0.107	0.093	0.160
pos258pos273	-0.020	-0.006	-0.001	0.000	0.014	0.056	0.020	-0.021
pos271pos273	0.155	0.109	0.142	0.120	0.162	-0.004	0.116	0.178
pos72pos193pos258	-0.005	0.037	0.007	0.010	0.001	0.027	-0.013	0.038
pos72pos193pos271	0.030	0.045	0.029	0.018	0.028	0.026	0.002	0.030
pos72pos193pos273	0.167	0.064	0.084	0.044	0.103	0.016	0.062	0.094
pos72pos258pos271	0.006	-0.002	0.058	0.033	0.042	0.022	0.008	-0.044
pos72pos258pos273	0.083	0.104	0.014	0.019	0.041	0.041	-0.007	0.059
pos72pos271pos273	0.142	0.124	0.060	0.073	0.098	0.045	0.031	0.054
pos193pos258pos271	-0.004	-0.007	-0.006	-0.009	-0.005	-0.015	-0.020	-0.032
pos193pos258pos273	0.017	0.014	-0.003	-0.011	-0.019	0.026	-0.001	-0.019
pos193pos271pos273	-0.025	-0.028	-0.010	-0.033	-0.013	0.011	-0.001	-0.015
pos258pos271pos273	0.072	0.041	0.052	-0.010	0.058	0.016	0.041	0.049
pos72pos193pos258pos271	-0.010	-0.001	-0.017	-0.024	-0.019	-0.024	-0.017	0.049
pos72pos193pos258pos273	-0.008	-0.007	-0.012	-0.005	-0.012	0.014	0.007	-0.005
pos72pos193pos271pos273	-0.001	-0.025	-0.004	-0.017	0.012	0.003	0.000	-0.005
pos72pos258pos271pos273	0.005	-0.028	-0.016	-0.014	0.006	-0.012	0.002	-0.004
pos193pos258pos271pos273	0.002	-0.019	0.004	-0.019	-0.032	0.005	-0.008	0.002
pos72pos193pos258pos271pos273	-0.022	-0.014	0.006	-0.020	-0.026	-0.012	-0.014	0.012

Supplemental Table 1: All mutational effects as determined by linear models (see **Methods**) across all metal environments tested. Epistatic interactions were determined up to full fifth-order interactions.

Terms	Zn	Cu	Mg	Ni	Co	Cd	Mn	Ca
pos72	0.13	0.10	0.018	0.020	0.20	0.035	0.012	0.12
pos193	0.14	0.29	0.12	0.15	0.13	0.120	0.11	0.13
pos258	0.16	0.008	0.42	0.45	0.22	0.333	0.57	0.28
pos271	0.084	0.037	0.060	0.12	0.14	-0.036	0.033	0.035
pos273	0.17	0.25	0.082	0.038	0.076	0.031	0.055	0.16
First Order Model	0.68	0.68	0.70	0.77	0.76	0.018	0.78	0.72
Second Order Model	0.21	0.19	0.25	0.18	0.17	-0.065	0.19	0.22
Third Order Model	0.094	0.11	0.048	0.042	0.06	0.056	0.022	0.04
Fourth Order Model	0.0003	0.005	0.002	0.006	0.004	-0.037	0.001	0.004
Fifth Order Model	0.0007	0.0006	0.0001	0.002	0.001	0.025	0.0006	0.0003

Supplemental Table 2: R^2 values for all linear models tested. These reflect the proportion of overall variation (including experimental error) that is explained by a linear relationship with the specified mutation or the respective model (non-epistatic = first order, pairwise epistasis only = second order, higher-level epistasis = third through fifth orders). For second-fifth order models the R^2 specified is the increased R^2 that results from fitting the more complex model as compared to the next highest-order model.[46]
